# Managing the potential and pitfalls during clinical translation of emerging stem cell therapies

**DOI:** 10.1186/2001-1326-3-10

**Published:** 2014-05-09

**Authors:** Heather Main, Megan Munsie, Michael D O’Connor

**Affiliations:** 1 The University of Sydney, Sydney, Australia; 2 Stem Cells Australia, Department of Anatomy and Neuroscience, The University of Melbourne, Sydney, Australia; 3 School of Medicine, University of Western Sydney, Campbelltown, Australia; 4 Molecular Medicine Research Group, University of Western Sydney, Campbelltown, Australia

**Keywords:** Pluripotent stem cell, Tissue specific stem cell, Clinical trial, Unproven treatment, Regenerative medicine, Autologous, Allogeneic, Homologous, Regulations

## Abstract

We are moving into a new era of stem cell research where many possibilities for treatment of degenerative, chronic and/or fatal diseases and injuries are becoming primed for clinical trial. These reports have led millions of people worldwide to hope that regenerative medicine is about to revolutionise biomedicine: either through transplantation of cells grown in the laboratory, or by finding ways to stimulate a patient’s intrinsic stem cells to repair diseased and damaged organs. While major contributions of stem cells to drug discovery, safety and efficacy testing, as well as modelling ‘diseases in a dish’ are also expected, it is the *in vivo* use of stem cells that has captured the general public’s attention. However, public misconceptions of stem cell potential and applications can leave patients vulnerable to the influences of profit driven entities selling unproven treatments without solid scientific basis or appropriate clinical testing or follow up. This review provides a brief history of stem cell clinical translation together with an overview of the properties, potential, and current clinical application of various stem cell types. In doing so it presents a clearer picture of the inherent risks and opportunities associated with stem cell research translation, and thus offers a framework to help realise invested expectations more quickly, safely and effectively.

## Review

The immense scientific, media and public interest in stem cells over the past 15 years has largely been focused on two areas: the therapeutic potential of stem cells, and ethical controversies relating to the destruction of human embryos historically required to obtain pluripotent stem cells. What is rarely fully appreciated is the significant research progress made during these years that has resulted in a large number of stem cell-based clinical trials currently, or soon to be, underway [1,2]. At the same time there has also been a rapid increase in the number and range of unproven stem cell therapies being offered worldwide. These unproven procedures are rarely underpinned by strong scientific evidence and are not tested through rigorous clinical trial assessment, thus making it difficult to support any claims made by the providers other than ‘selling hope’. Moreover, these unproven treatments are marketed directly to patients in an attempt to bypass the expert opinion of the patient’s treating physician(s). The emergence of unproven stem cell therapies alongside legitimate stem cell clinical trials neatly highlights the potential and pitfalls inherent within the stem cell field, both of which are driven by the desire of patients for desperately needed new treatments. Best management of these critically competing issues, to enable progress to be made as quickly as possible without exploitation of vulnerable patients, will be aided by a clear understanding of how the stem cell field has developed to where it is now.

### Stem cell properties underpin their clinical potential

Stem cells can be divided into two broad categories, tissue-specific (‘adult’) stem cells and pluripotent stem cells. Both of these stem cell types are able to make identical copies of themselves during cell division, a process termed ‘self-renewal’. Importantly, as stem cells self-renew they retain the ability to produce the more mature functional cell types of the body, such as nerve, blood, heart or cartilage cells, through processes broadly termed ‘differentiation’. Tissue-specific stem cells normally only give rise to cells of the tissue in which they are found, for example, blood stem cells only produce blood cells. In contrast, pluripotent stem cells can produce any cell type of the body. In terms of clinical applications, both tissue-specific and pluripotent stem cells can be used to generate cells for autologous (patient-sourced) and allogenic (donor-sourced) therapies.

**Figure 1:**
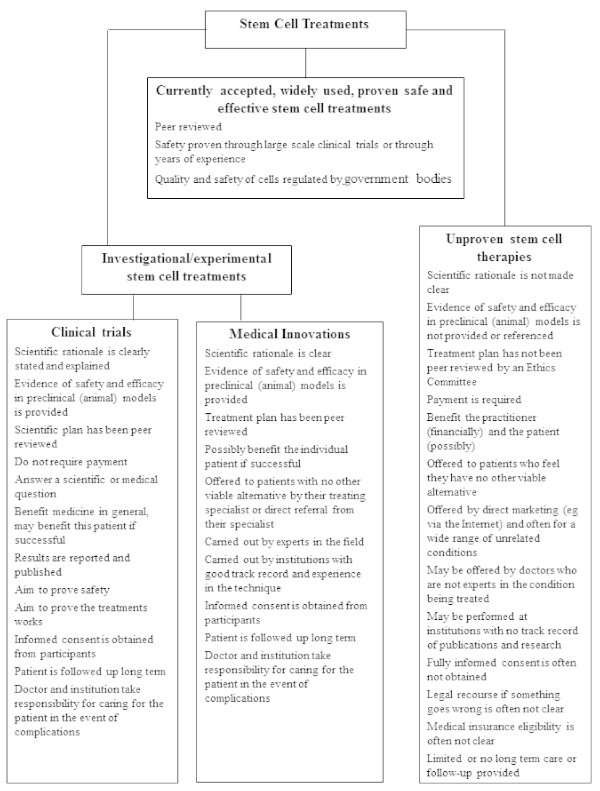
Key features that distinguish unproven stem cell therapies from other accepted cell treatments or investigations (Reproduced with permission from The Australian Stem Cell Handbook).

The development of clinical stem cell therapies has occurred progressively over the last 50 years, typically via the ‘medical innovations’ route (Figure 1). Commonly accepted clinical stem cell treatments are currently limited to therapies based on hematopoietic, skin, and corneal (limbal) stem cells. The first human hematopoietic transplant occurred in 1957 with the transplantation of bone marrow (containing blood stem cells) from one identical twin to the other in order to treat leukemia [3]. This work ultimately led to the 1990 Nobel Prize in Physiology or Medicine being awarded to Drs Murray and Donnall Thomas. However, progress from this first experimental human transplant to routine clinical therapy took decades of painstaking and publicly-reported research into the molecular mechanisms of the hematopoietic system. This included: discovery of human leukocyte antigen matching and its importance in minimization of graft-vs-host disease (GVHD) [4], and the discovery that hematopoietic stem cells (HSC) could be more easily obtained from peripheral blood (and subsequently cord blood) to provide less invasive and more efficacious transplant procedures [5]. As a result of these concerted international research efforts over 50,000 life-saving HSC transplants are now routinely performed worldwide every year [6].

The success of HSC transplants led scientists and clinicians to research stem cell-based therapies for other tissues. In 1981 skin stem cells were first harvested from burns patients, amplified in the laboratory and then transplanted back to the patient to partially reconstitute their skin [7]. The (limited) success of this initial procedure has led to a large number of skin grafts being performed since [8]. However, more basic research is needed to improve the quality of the resulting skin so that it possesses more characteristics of normal skin such as sweat glands and hair follicles [9,10].

In addition to saving lives, stem cell-based treatments have also been used to improve lives. In 1997 the first report was published showing that limbal stem or progenitor cells could be harvested from the edges of the cornea, proliferated in the laboratory and then transplanted back to patients to restore sight after corneal damage [11]. While improved cell harvest methods, cell expansion methods, and transplantation substrates are needed, the current limbal stem cell approach is capable of restoring sight for some forms of corneal disease and injury (collectively termed limbal stem cell deficiencies). As a result, this therapy has become widely used particularly in developing countries where corneal injury is more prevalent [12,13].

### Arrival of new cell sources for stem cell therapies

To scientists, doctors, journalists, policy makers and the public alike it appears self-evident that a primary driver behind the development of the above three stem cell treatments was a desire to treat patients with fatal or chronically debilitating conditions. However, it has to be recognized that their refinement into effective clinical therapies, through dramatic improvements in efficacy and reduced treatment invasiveness, arose as a direct result of extensive basic research. This research, and its wide and transparent dissemination, created testable hypotheses that were assessed in controlled clinical settings. For example, the successful application of HSC for lymphopenia, leukemias and lymphomas only became possible after extensive research provided a detailed molecular understanding of GVHD [3,4]. For skin and corneal grafts, targeted research into the identification, harvesting, expansion and transplantation of appropriate stem/progenitor cells was needed before the treatment of patients could be attempted [11,14]. Clearly, successful development of these clinical therapies hinged on continuous evidence-based research to improve treatment efficacy and safety, and to reduce patient discomfort.

As a result of the clinical achievements made using hematopoietic, skin and corneal tissue-specific stem cells, tremendous international research efforts have been initiated to identify stem cells in other organs of the body. Tissue-specific stem cells have thus been identified in most organs [15], helping not only explain the regenerative capacities of the body but providing new opportunities for the development of stem cell-based treatments for diseases and injuries of these tissues (Table 1). In particular mesenchymal stem or stromal cells (MSC) identified in a wide range of tissues (including adipose tissue, bone marrow, amniotic fluid, umbilical cord, placenta, menstrual blood and dental pulp) have generated a great deal of clinical interest. MSC have been shown in vitro to differentiate into adipocytes, chondrocytes, osteoblasts, and myocytes [16,17]. MSCs have also been shown to have immuno-modulatory properties that, dependant on the MSC source and treatment context, may demonstrate diverse effects from aiding tissue repair [18] to promoting tumour formation [19].

**Table 1:** Summary of some clinically relevant stem cell types and properties

**Stem cell categories**	**Examples of specific stem cell types**	**Stem cell sources**	**Properties/Challenges**
Pluripotent stem cells	Embryonic stem cells (ESCs) Induced pluripotent stem cells (iPSCs)	Surplus human IVF blastocysts (ESCs) Reprogrammed somatic cells (iPSCs)	Can produce any cell in the body (ESCs, iPSCs)
			Unlimited self-renewal capacity in vitro (ESCs, iPSCs)
			Difficult to generate pure populations of mature cells with current culture conditions (ESCs, iPSCs)
			Perceived ethical issues with some community groups (ESCs)
			Can be used for autologous (iPSC) or allogenic (ESC, iPSC) treatment
Tissue-specific stem cells	Hematopoietic stem cells (HSCs) Skin stem cells (SSCs) Mesenchymal stem cells (MSCs)	Bone marrow (HSCs, MSCs) Peripheral blood (HSCs)	Normally produce only cells from the particular tissue in which they are found
		Skin (SSCs)	Typically have limited self-renewal and expansion capacity in vitro with current culture conditions
		Fat (MSCs)	
		Cartilage (MSCs)	
			Difficult to generate pure populations of mature cells with current culture conditions
			Can be used for autologous or allogenic treatment

Due to the ease of MSC harvest (e.g., via liposuction), as well as their differentiation and immunomodulation properties, a large number of MSC-based clinical trials are progressing based on both their potential for pharmaceutical action or cell-replacement [2,20]. However, variations in protocols used for MSC purification, the lack of a definitive and universal MSC marker, and a poor molecular understanding of MSC action in disease settings currently make it difficult to interpret and compare much ‘MSC’ research and clinical data [21,22]. Efforts to overcome these limitations are underway, such as the proposed use of minimal criteria for characterization of MSCs in order to improve both intra- and inter-laboratory data comparisons [23].

Autologous (patient-sourced) transplantation or in vivo activation of tissue-specific stem cells offers an opportunity to develop therapies without requiring immunomodulation. However, historic and continuing difficulties in purifying, maintaining and/or expanding tissue-specific stem cells *in vitro* has hampered development of novel therapeutic approaches using these cells. Moreover, the fact that tissue-specific stem cells can each only make a limited number of mature cell types makes production of complex tissues from these cells challenging. To overcome these significant practical limitations of tissue-specific stem cells, alternative sources of stem cells with greater developmental potential have been investigated. As a result, a variety of methods have been found for producing human pluripotent stem cells that are capable of generating any cell type in the body. These include: embryonal carcinoma cells derived from teratocarcinomas [24]; embryonal germ cells [25]; embryonic stem cells derived from donated supernumery preimplantation blastocysts [26] or somatic cell nuclear transfer [27]; and, perhaps most remarkably, induced pluripotent stem cells typically created by introducing expression of four exogenous ‘reprogramming factors’ into nucleated somatic cells [28]. Importantly all of these human pluripotent stem cells, with the exception of embryonal carcinoma cells, can be maintained indefinitely in the laboratory with normal karyotype to provide a scalable source of normal (or diseased) human cells for research and clinical applications. The developmental potential, extensive proliferative ability, and economies of scale provided by human pluripotent stem cells confer enormous clinical potential to these cells (Table 1). However, the likely need for immunosuppression when transplanting their differentiated progeny provides a clear avenue for transplantation of autologous tissue-specific stem cells. As described in the next section, it remains to be seen which stem cell type (or types) will provide viable clinical therapies for particular diseases.

### Clinical trial of emerging stem cell therapies

**Figure 2:**
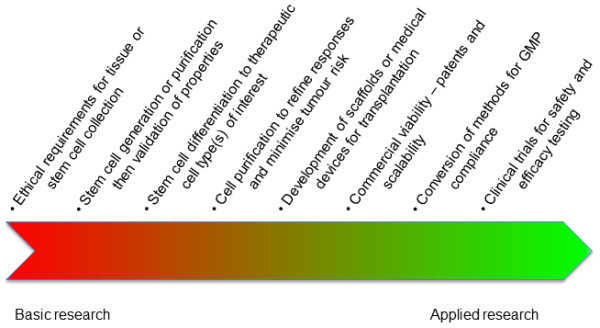
Schematic of the development pipeline for stem cell therapies.

Key to developing any new cell therapy from tissue-specific or pluripotent stem cells is to identify, purify and in some cases expand the most appropriate stem or differentiated cell type (Figure 2). At the same time unwanted side-effects, such as transplantation of inappropriate or tumorigenic cells, need to be avoided. This requirement presents a common challenge for both tissue-specific and pluripotent stem cells since iterative experimentation is required to: i) identify the optimal cell type for disease intervention, and ii) establish appropriate conditions to generate and purify a clinically useful number of the required cell(s) [29-33]. A range of related considerations (and their influencing factors) that need to be addressed during development of stem cell-based clinical trials are listed in Table 2.

**Table 2:** An indicative and interconnected list of some considerations to be addressed during the iterative clinical trial development process

*1. Transplant cells and method*: what disease stage should be targeted; what cell type(s) will produce the best outcome; what stem cell source is most appropriate for producing the cell of interest (i.e., tissue-specific or pluripotent); what cell formulation will produce the best outcome (single cell type, 3D tissue, +/- scaffold); where should the stem cell-derived cells be transplanted; how many cells or what sized tissue is needed; how should the transplant be delivered (directly to site or will cell homing be required); Good Manufacturing Practice manufacturing considerations; cost and timeframe for cell harvest, expansion, storage, transport and transplant preparation (do these fit the disease/injury repair timeframe); how to monitor/track the transplanted cells	*2. Autologous vs. allogenic cells*: are cells obtained directly from the patient (autologous) more appropriate than those from a donor (allogeneic); do appropriate methods exist for either stem cell source to generate sufficient purified cells for transplantation; cost and timeframe for cell harvest, expansion and transplant preparation (do these fit the timeframe for disease/injury response); likely complications of immune-rejection and/or immunosuppressive regimes; will autologous sources provide non-diseased cell types.	*3. Avoidance of tumorigenicity*: do the target transplant cells have intrinsic tumor forming potential; how to ensure residual unwanted cells with tumor forming potential are absent from the transplant (e.g., residual pluripotent stem cells); could the transplant environment induce aberrant proliferation of the transplanted cells; how will potential tumorigenicity be managed	*4. Navigating the regulatory environment*: where will the clinical trial be conducted; what regulations are involved; are patent licenses required; homologous vs. non-homologous cell applications (i.e., will the grafted cells perform exactly the same function as their endogenous normal counterparts or not); degree of manipulation of the cells ex vivo; relevance and risk associated with ‘fast-tracking’ approval of new treatments; ‘compassionate’ use and medical innovation	*5. Ethical oversight*: how will patients be recruited and at what stage of the disease; how will patients be informed about the cell type to be used, the expected scientific/mechanistic basis of the treatment and associated animal or human evidence for this; what consent will be required; what cells will be transplanted and how; how will treatment efficacy/side-effects be monitored and measured; what human research ethics committee is appropriate/required; what arrangements are in place to contend with adverse outcome and ongoing care; how will the patient’s condition be monitored; how will data be collected and shared	*6. Infrastructure and leadership*: cell therapy training and clinical expertise of those conducting the clinical research; need for specialist centres and equipment including Good Manufacturing Practice expertise and facilities; medical insurance to provide support in the advent of adverse outcomes; key leadership for integration of procedures; support personnel required for recruitment, patient counseling and clinical support; funding sources and cost-modeling of ultimate therapy; business case; milestones and go/no-go points

A recent review of the global landscape of stem cell clinical trials shows ‘novel’ applications - trials that do not involve established stem cell therapies - have grown dramatically, particularly using tissue-specific stem cells [2]. Stem cell companies, hospitals and research institutions are supporting cell-based clinical trials for treatment of stroke, myocardial infarction, inflammatory bowel disease, GVHD, diabetes, critical limb ischemia, spinal cord injury and macular degeneration. Many different stem cell sources, including fetal tissue, umbilical cord, and adipose tissue are being investigated, with the majority of the trials currently underway involving MSCs from various sources [2]. It should be noted, however, that due to the many unknowns surrounding MSC purification and mechanism described above, some leading stem cell clinician scientists have questioned the scientific basis and patient merits of some of these MSC trials [1,22].

In contrast, the first clinical trial to be approved worldwide using cells derived from human pluripotent stem cells received approval from the USA Food and Drug Administration (FDA) in 2010. This Phase I trial used human embryonic stem cell-derived oligodendrocyte precursor cells to treat acute spinal cord injury. Unfortunately, despite the immense effort invested in navigating the FDA regulatory requirements to establish this ‘first in man’ trial, in 2011 it fell victim to the high costs involved [34]. The company running the trial (Geron Corporation) made a controversial strategic decision to halt new enrolments, though the patients treated to this point continue to be monitored. This decision was made so the company could concentrate their resources on their anti-cancer platform. The decision came as a harsh blow to many researchers and patients who had been waiting for the opportunity to evaluate this new approach. It also raised important ethical questions such as the obligation to secure sufficient funds to complete a stem cell clinical trial.

Since commencement of the Geron trial two other human embryonic stem cell-based trials have begun and others are planned. The two commenced trials are using embryonic stem cell-derived retinal pigment epithelium to treat dry, age-related macular degeneration and Stargardt’s macular dystrophy [35]. In 2013 the trial expanded from the USA to Europe, though it is unclear from the short-term patient data whether the transplanted cells have objectively improved visual acuity in the treated patients [35,36]. Approval has also recently been obtained in Japan for the first clinical pilot study using retinal pigment epithelium derived from human induced pluripotent stem cells [37]. Rather than using one donor cell source to treat multiple patients (as with the human embryonic stem cell-based trials), this induced pluripotent stem cell trial will provide patients with *in vitro* produced autologous cells. Six patients will have biopsies taken for induced pluripotent stem cell production, from which retinal pigment epithelial cells will be differentiated, purified and seeded onto a cell substrate for transplantation. This process will be time, labour and cost intensive, with it expected to take 10 months before the grafts are ready for transplantation. Given that human induced pluripotent stem cells were only first described in 2007 [28] it is a remarkably short time to now be at the cusp of clinically testing these cells. However, it remains to be seen whether this type of highly technical and individualized patient-specific treatment is practical, effective and financially viable.

### Unmet patient needs and the emergence of unproven commercial therapies

The complexity of many incurable conditions, together with our insufficient understanding of normal and abnormal biology, means that it will be years before many who hope to benefit from stem cell therapies will be able to participate in clinical trials. Moreover, while hundreds of registered stem cell-based clinical trials are currently underway for a wide range of conditions [2], the majority of these are in the early-phase of testing to establish the safety of the proposed interventions. As a result, the number of patients able to participate is limited and there are stringent exclusion criteria. These issues mean that many patients feel they are denied access to treatment. Rather than waiting to participate in clinical trials or waiting for the outcomes of these trials, many patients are prepared to risk pursuing unproven stem cell treatments outside of clinical trials and often without scientific basis, adequate safety data, or ethical and regulatory scrutiny [38-40]. Promoted on impressive-looking websites, these unproven therapies often make use of moving patient testimonials to attract patients but have very little in the way of evidence to support the claimed benefits [41,42].

These unproven stem cell ‘treatments’ include the use of autologous and allogenic stem cell transplants, and are estimated to attract thousands of patients from around the world each year [43]. Until recently, these unproven treatments have typically involved travel to countries with less stringent regulation of experimental stem cell treatments. Such travel is often dubbed ‘stem cell tourism’ [44] and has raised great concern within the international stem cell community [45] due to the potential for real harm that may befall patients undertaking the treatments. Indeed, complications including meningitis [46], encephalomyelitis [47], tumours [48,49], aberrant bone formation [50] and at least two reported deaths [51,52] have all been attributed to unproven stem cell treatments.

More recently, there has been growth in the number of clinics selling treatments using a patient’s own cells in countries with considerable regulation such as Australia and the USA [53]. Regularly but inaccurately marketed as having ‘no risks’, being ‘natural’, or being accepted medical practice, these unproven therapies routinely lack credible scientific evidence or overstate/inappropriately extrapolate the significance of animal data or preliminary human studies. In stark contrast to the ground-breaking but experimental hematopoietic and skin stem cell treatments that first occurred in the 1950s and 1980s respectively, these modern commercially-marketed unproven therapies typically have little or no patient follow-up and provide no data to further international research efforts into treatment development.

Proponents of unproven commercial therapies argue that a patient should have the right to pursue treatment using their own cells and blame over-regulation for delaying clinical progress of stem cell treatments [54,55]. Even 2012 Nobel Prize winner in Physiology and Medicine, Sir John Gurdon, has criticised the FDA for placing "immense conditions on approval" saying "I think patients would be happy to take the risk of using their own cells given the choice" [56]. In contrast others are concerned that early adoption of unproven stem cell interventions, outside ethical review and the clinical trials processes, places individuals at risk of personal harm. Such unproven stem cell interventions also raise the significant risk of placebo effects being mistaken for genuine cures, and could stymie progress in the field. A recent statement from the International Society for Stem Cell Research (ISSCR) has urged ‘medical licensing bodies, legal authorities, patient advocacy organizations, physicians, and others to exercise their influence to discourage commercial provision of unproven autologous cell-based interventions outside of clinical trials’ [57].

### Evolving regulatory frameworks for stem cell therapies

Although there is still a role for assessment of innovative medical treatment using unproven stem cell interventions - like the initial 1957 hematopoietic and 1981 skin stem cell grafts - this approach should be considered as a ‘one-off’ and not offered widely or commercially until proven safe and effective. As recommended by the ISSCR this approach should only occur when certain conditions are satisfied such as: a robust scientific rationale; peer review of the proposed approach; data collection and clinical follow-up; undertaking only to treat a small number of patients who have provided informed consent; and commitment to more formal clinical trials in a timely manner (Figure 1).

Attempts to incorporate these recommendations while also decreasing the time for translating stem cell therapies are already underway. For example, recent legislation has provided the USA FDA with a new breakthrough therapy designation that offers the possibility of accelerated approval for investigational new drugs for serious conditions [54]. In late 2013 Japan approved two new pieces of legislation: one that requires physicians to report the use of unapproved stem cell therapies to the health ministry, and another that creates a new approval channel for regenerative medicine and stem cell therapies [58,59]. This new approval process appears similar to one used in South Korea, and also appears to be a variation of the currently accepted ‘medical innovations’ route of therapeutic development (Figure 1). At this stage though, a variety of important issues remain unclear such as: whether it will only be used to offer therapies to patients with no other viable alternatives; who will take responsibility for adverse outcomes; and what level of expertise will be required by the treatment provider.

This new Japanese legislation will enable companies to initiate small pilot treatment studies with as few as 10 patients without having to perform traditional Phase III clinical trials. If some degree of efficacy is shown in the pilot study, then the therapy can be approved for commercial use for 5 to 7 years, with possible coverage through the national health system. Once permission for commercialisation has been granted all patient data must be captured in a treatment registry, and approval can be withdrawn if therapies prove unsafe or ineffective.

While this new approach may reduce the risk of harmful side-effects from emerging and unproven stem cell therapies, it nevertheless has the potential to promote the sale of unproven and potentially costly stem cell therapies to desperate and vulnerable patients. It also raises the potential for patients to bypass currently available and effective treatments in favour of directly-marketed unproven therapies. It may also lead to misleading placebo effects becoming ingrained within the field since commercial treatments cannot be supplied, and their efficacy tested, as done with traditional double-blind clinical trials. Additionally, should any therapy commercialised in this way prove ineffective (or worse, identify previously unknown harmful side-effects), then large numbers of patients will have been subjected to inappropriate risks associated with medical procedures at significant health, financial and emotional cost.

## Conclusions

The pace of research into tissue-specific and pluripotent stem cells continues to increase, with new advances announced regularly. In this review we see that solid foundations of basic molecular research are required, not necessarily for establishment of therapeutic concepts, but for development of therapies towards safe and effective clinical treatments. From less successful applications we see the dangers of unproven procedures and the challenges in developing therapeutic regimes with limited molecular understanding. As the appearance of stem cell-related clinical trials accelerates, failed trials and possibly unforeseen side-effects are likely to follow. At the same time there is a danger of harmful or fatal consequences resulting from the emergence of profit-driven unproven stem cell therapies that take advantage of vulnerable patients. A concerted effort is therefore required to identify pitfalls and safeguards for timely and effective clinical translation of emerging stem cell therapies. Additionally it is not yet clear which stem cell types will be best suited to treat particular diseases. Ultimately, only further empirical research will determine for each disease whether optimal treatment is provided by the extensive proliferative and differentiation potential of pluripotent stem cells, or the potential autologous nature of tissue-specific stem cells.

To address these issues we need to continue to diligently manage the expectation of patients and their families and friends, as well as those of the media, governments and financiers wanting to invest in the area. This includes correcting the misunderstanding that any source of stem cells is an automatic and unquestioned panacea for all ailments.

We need to accelerate access for patients to well-regulated and adequately funded clinical trials, but these trials need to be based on a detailed, mechanistic understanding of the disease process with strong supporting pre-clinical data. We must also acknowledge that it is going to take time to develop new stem cell treatments. This will help to avoid academic, social and economic pressures from eroding the integrity of, and community confidence in, the regenerative medicine field. The recent controversy surrounding the Stimulus-Triggered Activation of Pluripotency (STAP) method [60] highlights this need, as well as the need for robust and transparent oversight processes at all levels, from basic research through to clinical translation.

We need to share results from all clinical research so that further research can be undertaken to evolve or develop promising new clinical therapies. We need to provide opportunities for innovative medicine on compassionate grounds but prevent over-enthusiasts from providing unproven and potentially unsafe procedures. Enforceable frameworks need to be developed that ensure new or ‘novel’ stem cell treatments are at least first proven safe through independent, peer-reviewed Phase I and II clinical trials in order to define for the patient the severity and frequency of unwanted side-effects.

We must ensure that any new therapy is provided only with informed patient consent and rigorous long-term post-treatment monitoring. We recommend that any commercially available stem cell therapies not assessed via Phase III clinical trials should be required to be designated as experimental treatments. It is only through recognising and addressing these issues that the field will safely progress at a rate closer to the expectations of patients, thus realising maximal patient benefits without simply ‘selling hope’.

## Abbreviations

ESC: Embryonic stem cells; FDA: Food and drug administration; GVHD: Graft-vs-host disease; HSC: Hematopoietic stem cells; ISSCR: International society for stem cell research; IVF: In vitro fertilization; MSC: Mesenchymal stem cell.

## Competing interests

The authors declare that they have no competing interests.

## Authors’ contributions

The manuscript was conceived by MO’C with valuable input from MM and HM. HM, MM and MO’C all contributed to writing and editing the manuscript. All authors read and approved the final manuscript.
